# Is a *Plasmodium* lactate dehydrogenase (pLDH) enzyme-linked immunosorbent (ELISA)-based assay a valid tool for detecting risky malaria blood donations in Africa?

**DOI:** 10.1186/1475-2875-12-279

**Published:** 2013-08-08

**Authors:** Pascal S Atchade, Cécile Doderer-Lang, Nicodème Chabi, Sylvie Perrotey, Tamer Abdelrahman, Casimir D Akpovi, Ludovic Anani, André Bigot, Ambaliou Sanni, Ermanno Candolfi

**Affiliations:** 1 Institut de Parasitologie et de Pathologie Tropicale (IPPTS) - Fédération de Médecine Translationnelle, Université de Strasbourg, Strasbourg, France; 2 Laboratoire de Biochimie et de Biologie Moléculaire, Université d’Abomey Calavi, 04 BP 0320 Cotonou, Bénin; 3 Public Health Wales Microbiology, Public Health Wales NHS Trust, Cardiff, UK; 4 Agence Nationale pour la Transfusion Sanguine (Ministère de la Santé), 01 B.P. 511 Cotonou, Bénin

**Keywords:** Malaria, Transfusion, *Plasmodium falciparum*, pLDH, Antibodies

## Abstract

**Background:**

Malaria is a leading cause of mortality in southern Benin. The main causative agent, *Plasmodium falciparum*, poses a threat on critical transfusions in pregnant women and children. This study’s objective was to compare the performance of different malaria screening methods in blood donors in southern Benin, a malaria-endemic country.

**Methods:**

Blood from 2,515 voluntary blood donors in Benin was collected over a period of 10 months in ethylenediaminetetraacetic acid (EDTA) tubes, which were then classified according to extraction time: long rainy season, short dry season, short rainy season, and long dry season. Microscopic examination was used to count parasites. Parasite density (PD) was expressed as the number of parasites per μL of blood. Pan *Plasmodium* pLDH detection was assessed by an ELISA-malaria antigen test. Using crude soluble *P*. *falciparum* antigens*,* an ELISA-malaria antibody test detected anti-*Plasmodium* antibodies.

**Results:**

Among the 2,515 blood donors (2,025 males and 488 females) screened, the rate of asymptomatic *Plasmodium* carriage was 295/2,515 (11.72%, 95% CI: 10.5-13.1%). Males had a higher infection rate (12.4%) than did females (8.8%). Parasite density was very low: between seven and100 parasites per μL of blood was reported in 80% of donors with parasitaemia. Three *Plasmodium* species were diagnosed: *P. falciparum* in 280/295 patients (95.0%), *Plasmodium malariae* in 14/295 (5.0%), and *Plasmodium ovale* in 1/295 (0.34%). Malaria prevalence in donors was higher during the rainy seasons (13.7%) compared with the dry seasons (9.9%). The use of a highly sensitive assay enabled pan *Plasmodium* pLDH detection in 966/2,515 (38.4%, 95% CI: 36.5%-40.3%). Malaria antibody prevalence was 1,859/2,515 (73.9%, 95% CI: 72.16-75.6%). Donors’ antigenaemia and antibody levels varied significantly (P <0.05) over the course of the four seasons. The highest antigenaemia rate 323/630 (51.3%), was observed during the short rainy season, while the highest antibody prevalence, 751/886 (84.7%), was recorded during the long dry season.

**Conclusion:**

Blood donations infected with *Plasmodium* can transmit malaria to donation recipients. Malaria diagnostic methods are currently available, but the feasibility criteria for mass screening in endemic areas become preponderant. Detection of the pLDH antigen seems to be an adequate screening tool in endemic areas, for this antigen indicates parasite presence. Routine screening of all donated blood would prevent infected blood donations and reduce *P. falciparum* transmission in critical patients, such as children and pregnant women. This tool would also decrease medical prophylaxis in donation recipients and contribute to lower *Plasmodium* resistance.

## Background

Transfusion-induced malaria was first reported in 1911 [1], and it is well-established that all five human malaria parasites (*Plasmodium falciparum*, *Plasmodium malariae*, *Plasmodium vivax, Plasmodium ovale,* and *Plasmodium knowlesi*) may be transmitted via blood transfusion [2]. Blood is mainly used in the emergency management of patients with life-threatening anaemia accompanied by severe malaria and malnutrition [3]. Blood transmission of malaria is a potentially serious complication that poses a continuous risk for blood banks, as the recipient’s malaria diagnosis is not expected, and is thus often missed [3,4]. As malaria parasites can survive in red blood cells at refrigerator temperature (2-4°C) for days or weeks [5], this would require the exclusion of all blood donors with a potential risk [6,7]. Transfusion-transmitted malaria (TTM) is an important public health problem that affects most parts of the world. Over the past decade, TTM has been reported in low endemic countries, such as France [8], Brazil [9], the USA [10], and the UK [11]. The situation is more alarming in highly endemic areas, such as sub-Saharan Africa, where *Plasmodium* prevalence among blood donors may reach record levels of 51.50% [12,13] using a technique less sensitive than microscopy [14].

In Benin, as in other tropical developing countries, the high demand for blood donations due to increased road accidents, pregnancy-related haemorrhages and child anaemia enhances the risk of TTM. Benin’s humid tropical climate, which has two rainy and two dry seasons, favours malaria transmission over the course of eight months, with 58 infectious mosquito bites per man per year [15]. The most effective malaria vector, *Anopheles gambiae*, is the most widespread and difficult to control [16]. The majority of malaria-related deaths in Benin is caused by the lethal *P. falciparum*. In Benin, 37% of adult health facility visits and 41% of those by children under five years old are malaria-related. Malaria is the leading disease affecting communities, with pregnant women and children under five years old being the most vulnerable. The incidence of uncomplicated and severe malaria was 139 per 1,000 inhabitants in 2006 [17]. Self-medication for malaria prevention is likely to complicate these figures: although widespread in Benin, it has never been objectively assessed. TTM is not considered a priority in malaria-endemic countries, but malaria prevalence is nonetheless decreasing thanks to the Roll Back Malaria programme [18]. The significant risk of *Plasmodium* transfusion is expected to increase over the next few years in unprotected patients, such as pregnant women or children. Blood transfusion is the third transmission path of *Plasmodium*, and there is still no consensus on TTM preventative measures to be taken in tropical settings [19]. Malaria symptoms may be subtle or non-existent in many blood donors carrying *Plasmodium*[7], so screening questionnaires have limitations [20]. Moreover, routine malaria diagnosis is made by microscopic visualization of parasites on thick and thin smears, but this method is inadequate for examining a large volume of samples [14,20]. Nevertheless, microscopy is still commonly used in studies investigating malaria prevalence in blood donors [13,21-23]. The use of malaria antibody detection, as in non-endemic countries, is inadequate due the frequent high seroprevalence in endemic countries. As previously suggested, malaria antigen detection may be a feasible solution [7,19]. Therefore, this study’s objective was to assess the performance of a commercially available pLDH antigen detection ELISA-based assay compared to microscopy in 2,515 voluntary blood donors who were asymptomatic for malaria. Malaria antibody prevalence was also assessed.

## Methods

### Study sites and ethical consideration

Benin is characterized by tropical rainforest vegetation, Sudanese climate type with an annual rainfall of about 1,600 mm, and an atmospheric temperature of 32°C. There are four distinct seasons: two rainy and two dry. This descriptive, transversal study was conducted in Benin over ten consecutive months in six departments, which grouped into three pairs of Departmental Blood Transfusion Centres (DBTC): Atlantique-Littoral,Oueme-Plateau, and Mono-Couffo. They serve as referral centres for south Benin blood transfusion services. This study was approved by the Direction of Benin National Blood Transfusion Agency and the Research Ethics Committee of the Republic of Benin. Approval was granted under the following conditions: donor anonymity must be maintained, good laboratory practice quality control must be ensured, and every finding must be treated with utmost confidentiality and used only for this research purpose. Biological analyses of the samples were performed in the biochemistry and molecular biology laboratory in Cotonou (Benin) and the Institut de Parasitologie et de Pathologie Tropicale de Strasbourg (IPPTS) (France).

### Study population

Individuals considered healthy who visited the three DBTCs for blood donation between May 2009 and March 2010 were considered eligible for the study. A total of 2,515 donors and regular voluntary blood donors were enrolled over a period of ten months, which was divided into a long rainy season (LRS) from May to July, a short dry season (SDS) from August to September, a short rainy season (SRS) from October to November, and a long dry season (LDS) from December to March. Donors were selected according to medical screening criteria: no fever, no clinical evidence of progressive disease, weight above 50 kg, age between 18 and 60 years old, or 65 years old for regular donors. Last blood donation had to have occurred within the previous four months for women and three months for men. A more systematic questionnaire was introduced providing the full name, age, sex, address, and telephone of each individual. Malaria-related questions were also included: date and confirmation via clinical manifestation of malaria episodes that occurred during the year, subsequent treatment and prevention measures taken against malaria. Each study participant signed an informed consent. Overall, 7 mL of blood were collected in EDTA tubes, with 1 mL transferred into a microtube and the remaining 6 mL centrifuged at 4°C, plasma being collected into a new microtube. The tubes were stored at −20°C and transported to the IPPTS laboratory in solid carbon dioxide for antibody and pLDH antigen detection.

### Parasitological examination

A thick and a thin smear were performed on slides for each Benin blood donor. The thick film was dehaemoglobinized using distilled water at pH 7.2, and then thick and thin blood films were stained using a May-Grünwald-Giemsa (MGG) stain. The microscopic reading and parasite density calculation were performed by two laboratory technicians, and in the case of uncertainty, also by a third one. Parasite count was made on the basis of 500 parasites per 1,000 leukocytes. The reading was stopped after 500 parasites were counted, even though the 1,000 figures of leukocytes were not reached. Parasite density (PD) was expressed as the number of parasites per μL of blood.

### pLDH antigen detection

pLDH is a glycolytic pathway enzyme secreted by the different *Plasmodium* species, but it possesses species-specific isomers [24]. The pLDH enzyme disappears within 24 hours of effective malaria treatment [25]. Therefore, the pLDH antigen is considered a specific marker for the presence of viable *Plasmodium* in blood, and is used for screening in malaria-endemic countries. The pLDH antigen detection was performed by a sandwich enzyme-linked immunosorbent assay (ELISA), notably an ELISA-malaria antigen test (apDianv, Belgium) that detects pLDH via immunocapture. The apDia Antigen ELISA is an *in vitro* diagnostic immunoassay (IVD) for the qualitative determination of *Plasmodium* spp. LDH in blood samples. The apDia Malaria antigen test can be used in order to detect the malaria pLDH antigen of any of the four species in blood samples. The test was performed according to manufacturer recommendations: pour 100 μL of ready-to-use lysing buffer into each well; add 50 μL of reconstituted positive control to one well and 50 μL of negative control to triplicate well; add 50 μL of homogenized fresh whole blood sample into corresponding well; incubate for 60 min at 37°C under continuous gentle shaking conditions; drain the wells via aspiration, and fill them completely with 350 μL of washing solution; allow the wells to soak for 1 min before washing again five times; pour 100 μL of conjugate 1 solution into each well, and incubate the plate for 30 min at 37°C; wash the wells five times and pour 100 μL of conjugate 2 into each wells. Incubate the plate for 15 min then wash the wells five times and pour 100 μL of chromogenic solution into each well; incubate the plate for 15 min at 37°C; add 50 μL of stopping solution to all wells and read absorbance of each well at 450 nm with reference wavelength of 620 nm within 15 min. Test results were interpreted as follows: the optical densities of positive control (ODpos) must be >0.500 and the average OD of negative control (ODneg) <0.100. The ODneg was used to calculate the cut-off by multiplying its average value by three. The antigen index (Ag Index) of each sample was calculated by dividing the OD value of the sample by the cut-off value. A sample was considered positive if the Ag Index was ≥1.0, indicating that the sample contained viable parasites. A sample was considered negative if the Ag Index was ≤0.8, indicating that there were no viable parasites in the blood, or that there was no *Plasmodium* multiplication because of anti-malarial drug intake. A sample was considered inconclusive if the Ag Index was between 0.8 and 1.

### Measurement of sensitivity, specificity and detectability of the ELISA-based pLDH detection assay

#### Samples from malaria patients for sensitivity calculation

The whole blood biobank collected from 266 malaria patients who had returned from endemic countries, positively diagnosed and referred by Strasbourg University hospital was tested. Among them, 239 were positive for *P. falciparum*, 19 for *P. ovale*, six for *P. malariae,* and two exhibited a mixed infection (*P. ovale* and *P. falciparum*). This panel was used to assess the sensitivity of the ELISA antigen detection test compared to microscopy.

#### Samples from blood donors for specificity calculation

Blood donor samples were collected at the French Etablissement Français du Sang d’Alsace (EFS Alsace). Based on a medical questionnaire, donors were classified as “malaria-risk blood donors” (n=1,771) if they had travelled to an endemic area in the previous four months, or “not-exposed-to-malaria blood donors” (n=1,781) if they had not travelled to an endemic area in the previous three years.

### Cultured parasites

The 3D7 strain of *P. falciparum* was maintained *in vitro* using an adapted candle jar method for continuous culture [26]. Parasites were cultured in normal O-positive red blood cells (RBCs) from healthy donors (EFS Alsace) and malaria culture medium (MCM, pH 7.4) composed of Roswell Park Memorial Institute medium (RPMI) 1640 supplemented with 2 mM L-glutamine, 10 mM Hepes (Gibco, Invitrogen, Cergy Pontoise, France), 1 μg/ml hypoxanthine, 0.11 mg/ml Na pyruvate, 0.02 mg/ml gentamycin, and 10% (V/V) alpha calf serum (Perbio Science, Brebières, France). The culture was diluted at a parasitaemia and haematocrit of 1%, and the medium was changed every 48 hours.

### Determination of parasite detectability threshold using microscopy and pLDH Elisa test

Culture with 65% ring-stage parasites and 35% schizonts was used. Infected RBCs from the continuous culture at 1% parasitaemia determined on thin blood film by microscopy were serially diluted in uninfected RBCs by incubation with low agitation at 37°C. Dilution ranged from 20,000 infected RBCs (iRBCs)/μl to 0.1 iRBCs/μl. For microscopy examination, thin blood films were made from each dilution after careful suspension, while thick blood films were made for the lower parasitaemia. Three slides were prepared for each dilution and read by two microscopists in a blind manner. Parasitaemia was determined by counting fields with 100 RBCs, and the total level was expressed in μL of blood corresponding of 5.106 RBCs/μL. A linear regression curve based on mean parasitaemia levels was established in order to determine the parasitaemia, and a standard curve was generated using recombinant pLDH to assess the correlation between parasitaemia and pLDH concentration.

### Recombinant pLDH

A synthetic gene (Genscript, NJ, USA) encoding for *P. falciparum* pLDH protein was inserted in the expression vector pMAL C2X (New England Biolabs, Ipswich, MA, USA) containing the maltose binding protein (MBP) fusion partner sequence. The protein was expressed in *Escherichia coli* and purified by affinity chromatography on amylose resin. The protein was used to generate the standard curve.

### Antibodies detection

Antibody screening was performed using a homemade ELISA-antibody test. The serological ELISA test was derived from a commercial assay exhibiting high specificity and analytical sensitivity, namely 96.7 and 93.1%, respectively [27]. The test was based on binding of anti-*Plasmodium* antibodies present in serum or plasma samples to antigens immobilized on 96-well plates, with the antigen being an extract of *in vitro P. falciparum* culture (3D7 strain*)*. Due to antigenic community, antibodies to other *Plasmodium* species may be detected. The diluent solution containing PBS and Tween 0.1%, (125 μL) was poured into each well, followed by 25 μL of test plasma. On the same plate, 25 μL positive control and negative control were poured in single and triplicate wells, respectively. The plate incubated for 60 min at 37°C before being washed five times. One hundred μL of horseradish peroxidase-conjugated rabbit anti-human IgG polyclonal antibodies (Sigma-Aldrich, St Quentin, France) were added to each well, and the plate incubated for a further 30 min at 37°C. The wells were washed five times, and 100 μL of TMB plus substrate solution (tetramethylbenzidine) (Kem-en-tec, Denmark) were added to each well. The plate was covered and incubated in the dark for 15 min at 37°C. Finally, 50 μL of 0.5 M sulphuric acid was added to each well, and absorbance was read within 15 min at 450 nm, with a reference wave-length of 620 nm. Test validation required that the positive OD be >0.500 and the negative OD≥0.200. The cut-off value was calculated by multiplying the negative control wells’ average OD by four. The antibody (Ab) index of each sample was calculated by dividing its OD value by the cut-off value. The sample was considered positive if the Ab index was >1.0, equivocal if the Ab index was between 0.8 and 1.0, and negative if the Ab index was ≤0.8. The test was not able to distinguish between antibodies directed to either *P. falciparum*, *P. vivax*, *P. malariae,* or *P. ovale*.

### Data analysis

The data was analysed by the chi-squared, Bartlett's chi-squared, and Kruskal-Wallis tests using Sigma Plot 2001 software (Sigma Plot 2001 Statistical Analysis Software, San Jose, CA, USA) and Epi Info version 3.5.1. The results were expressed as mean ± standard deviation. Statistical significance was reached if P <0.05, with a 95% confidence interval (CI).

## Results

### pLDH ELISA performance

#### Sensitivity

Among the 266 malaria patients, the antigen detection test was able to detect 250 cases while missing 16, resulting in a sensitivity of 94%, with borderline results included as positive in the calculations. Nine cases of *P. falciparum* were missed, five of them were treated for malaria before the blood examination and the four remaining patients did not provide any information’s regarding a previous treatment. Seven cases of *P. ovale* were also missed may be due to the fact that the monoclonal antibodies for pLDH fail to detect some *P. ovale* strains. However this point was not explored in our study and not discussed. The antibody detection test was able to detect only 220 cases (Table 1). Moreover, the combined tests detected 261 positive cases and missed only five, namely a *P. falciparum-*infected patient with prior anti-malarial treatment*,* and four *P. ovale-*infected patients (Table 2). Specificity for malaria-risk blood donors and not-exposed-to-malaria blood donors was 99.6 and 99.5%, respectively, with an overall specificity of 99.57%. The pLDH assay’s positive and negative predictive values were 94.3% and 99.5%, respectively.

**Table 1:** pLDH ELISA and Malaria antibody ELISA results in a population of microscopically confirmed malaria patients (n= 266)

**Species**	**Microscopy**	**pLDH ELISA**	**Antibody ELISA**
***P. falciparum***	239/266 (89.9%)	230/239	199/239
***P. ovale***	19/266 (7.1%)	12/19	14/19
***P. malariae***	6/266 (2.25%)	6/6	5/6
**Mixed infection**^**1**^	2/266 (0.75%)	2/2	2/2
Total		250/266 (94%)	220/266 (82.7%)

^1^Mixed infections are *P. ovale* with *P. falciparum*.

**Table 2:** Comparison of pLDH ELISA (Ag) and antibody ELISA (Ab) detection in a population of microscopically confirmed malaria patients (n= 266)

	**Ab Pos**	**Ab Neg**	**Total**
Ag pos	209	41	**250**
Ag neg	11	5*	**16**
TOTAL	**220**	**46**	**266**

*One case of *P. falciparum* infection treated prior to the parasitological diagnosis and four *P. ovale* cases.

#### Detectability

**Figure 1:**
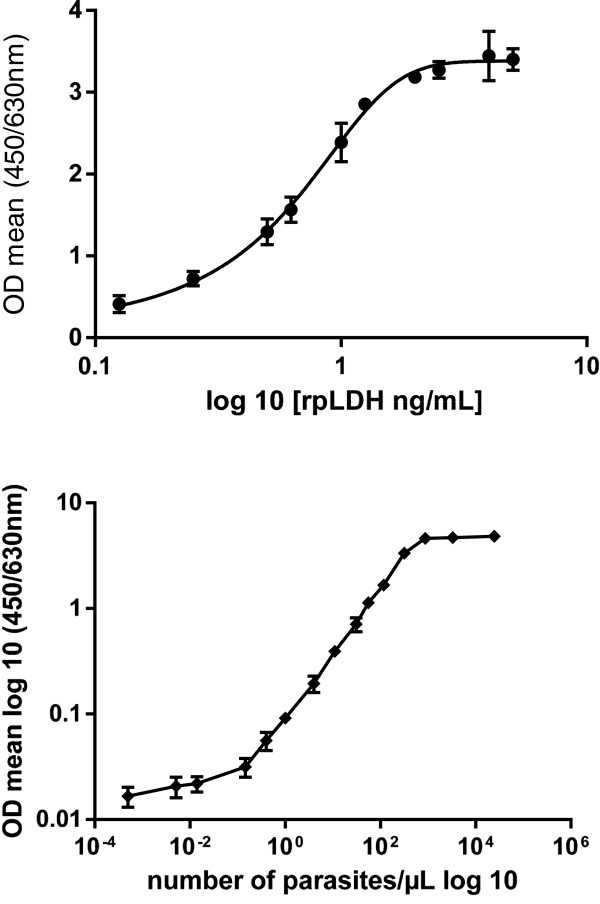
**pLDH detectability for *Plasmodium falciparum *and recombinant pLDH.** Standard curve for a range of ten assays for rpLDH and 15 assays for Pf iRBCs. Detectability was 2.5 ng/mL for rpLDH and one parasite per μL for RBC.

The parasite detection threshold for microscopy and pLDH Elisa test was calculated using a range of infected iRBCs and recombinant pLDH. The calculated parasitaemia in diluted iRBCs ranged from 24,676 iRBCs/μl to 0.0005,iRBCs/μl (extrapolated value), with a linear regression line and 0.977 correlation coefficient. The detection limit was 1 parasite/μL for the pLDH ELISA, which corresponds to 0.08 ng/ml of recombinant pLDH. The detection threshold with the standard curve using the recombinant pLDH was 0.125 ng/mL, the curve being linear up to 2.5 ng/mL (Figure 1) (Table 3).

**Table 3:** **Detectability of pLDH evaluated with a recombinant pLDH (A) and *Plasmodium falciparum*-infected red blood cells (*Pf *iRBCs) (B)**

**A**		
**rpLDH ng/mL**	**Index**	**SD**
0	0.335	0.071
0.125	1.465	0.365
0.25	2.650	0.312
0.5	4.595	0.553
0.625	5.550	0.550
1	8.464	0.830
1.25	10.129	0.149
2	11.299	0.145
2.5	11.607	0.358
4	12.11	1.067
5	12.057	0.461
**B**		
**PARASITE/μL**	**Index**	**SD**
0	0.194	0.061
0.0005	0.165	0.036
0.0051	0.205	0.045
0.0141	0.217	0.036
0.1455	0.312	0.064
0.4	0.556	0.110
1	0.908	0.100
4	1.922	0.335
11	3.886	0.310
31	7.017	1.073
56	11.227	1.000
117	16.523	1.609
319	33.032	3.073
872	45.664	2.435
3,298	46.396	2.353
24,676	47.630	2.444

### pLDH assay evaluation in a population of Benin blood donors

#### Characteristics of the blood donor population

Blood samples from 2,515 donors were collected over a ten-month period covering four seasons: LRS, SDS, SRS, and LDS. The samples were distributed as follows: 590/2,515 (23.5%) in LRS, 409/2,515 (16.3%) in SDS, 630/2,515 (25.0%) in SRS and 886/2,515 (35.2%) (Table 4). The donors enrolled in this study were young, with a median age of 31±9 years. Their age distribution showed that young people between 25 and 40 years were the most represented, with 1,379/2,515 (54.8%). Sex distribution exhibited a strong male predominance with 2,027/2,515 (80.60%) men *versus* 488/2,515 (19.40%, 95% CI: 17.9-21.0%) women. Among the donors, 1,989/2,515 slept under a mosquito net (79.1%, 95% CI: 77.4-80.6%). The majority of donors, namely 1,521/2,515 (60.47%, 95% CI: 57.4-61.2%), reported having had episodes of malaria, and the predominant clinical prior malaria expression was a single suspected access in 1,387/2,515 (55.1%, 95% CI: 53.2-57.1%), whereas 51 donors (2.0%, 95% CI: 1.5-2.7%) reported a severe attack requiring hospitalization in the last year. In total, 83 donors (3.3%, 95% CI: 2.7-4.1) had both single and severe malaria attacks, whereas 994 donors (39.52%) reported having no malaria attack during the preceding year. Among the 1,521 donors with suspected malaria episodes, anti-malarial treatment was administered to 509 (33.46%) in health facilities during the last malaria attack, whereas 1,012/1,521 (66.53%) were treated as outpatients. Most blood donors did not receive chemoprophylaxis but performed a sort of prevention when they experienced “signs of malaria”. Self-prevention against malaria was undertaken by 1,537/2,515 donors (61.11%) who took anti-malarial drugs or herbal tea, whereas 978/2,515 (38.88%) did not undertake any prevention. The drugs used were quinine 567/2,515 (22.5%, 95% CI: 20.9-24.2%), artemisinin-based combination 510/2,515 (20.3%, 95% CI: 18.7-21.9%), herbal teas 336/2,515 (13.4%, 95% CI: 12.1-14.8%), and Fansidar® 124/2,515 (4.9%, 95% CI: 4.1-5.9%).

**Table 4:** **Distribution of microscopically detected *Plasmodium *species in a population of 2,515 blood donors during the four seasons: long rainy season (LRS), short dry season (SDS), short rainy season (SRS), and long dry season (LDS)**

**Sampling period**	**Positive/donors**	**Positivity rate**	**Prevalence species**
	**(N =2515)**	**(%)**	**(N= 295)**
**LRS** (May-June-July)	67/590	11.4	*P. falciparum* (64/67) 95.52%; *P. malariae (3/67) 4.47%; *P. ovale* (0) 0.0%
**SDS** (August -September)	48/409	11.7	*P. falciparum* (45/48) 93.75%; *P. malariae (3/48) 6.25%; *P. ovale* (0) 0.0%
**SRS** (October-November)	100/630	16.0	*P. falciparum* (96/100) 96.0%; *P. malariae (3/100) 3.0%; *P. ovale (1/100) 1.0%
**LDS** (December-January-February)	80/886	9.0	*P. falciparum* (75/79 (93.75%; *P. malariae (5/80) 6.25%; *P. ovale* (0) 0.00%
**TOTAL**	295/2,515	11.72	*P. falciparum* (280/295) 95.0%; *P. malariae (14/295) 5.0%; *P. ovale (1/295) 0.34% Mixed infection (15/295) 5.10%

*Mixed infection.

### Microscopic prevalence of *Plasmodium*

Among the 2,515 blood donors, the rate of asymptomatic *Plasmodium* carriage measured by microscopy was 295/2,515 (11.72%, 95% CI: 10.5-13.1%). Three *Plasmodium* species were detected: *P. falciparum* was identified in all infection cases, *P. malariae* in 14 mixed infection cases (5.1%), and *P. ovale* in one mixed infection case (0.34%) (Table 4). The study findings revealed a higher percentage of infection among males 252/295 (85.42%) compared to females 43/295 (14.6%). Microscopic prevalence varied according to seasons: *P. falciparum* and *P. malariae* detection was constant during all seasons (Table 4), whereas *P. falciparum* density was very low and highly variable. Among the 295 positive donors, 238 (80.67%) exhibited a parasite density between seven and 100 parasites per microliter (p/μL), with 0.3% exhibiting a density >5,000 p/μL (Table 5).

**Table 5:** Variation of parasitaemia in the 295 positive donors by microscopy

**P/μL**	**Frequency**	**Percent (%)**
7-100	238	80.7
101-500	1	15.9
501–5,000	9	3.1
> 5,000	1	0.3
**Total**	**295**	

### Malaria antibodies prevalence

**Figure 2:**
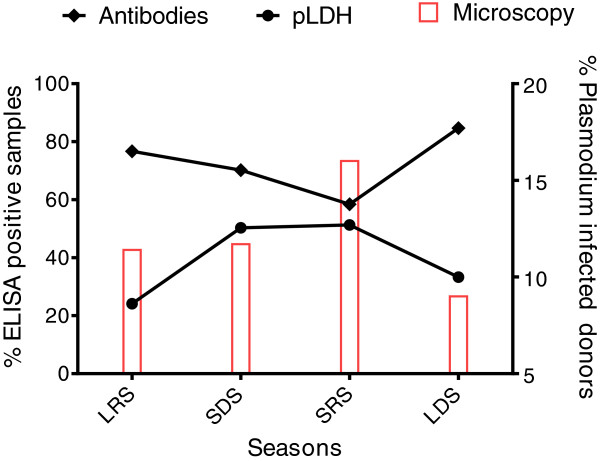
**Prevalence rates were calculated for *Plasmodium *presence by microscopy and pLDH detection, and for malaria antibodies in a population of asymptomatic blood donors (n= 2,515) over a ten-month period, divided in long rainy season (LRS) from May to July 590/2,515, short dry season (SDS) from August to September 409/2,515, short rainy season (SRS) from October to November 100/630, and long dry season (LDS) from December to February 80/886.**

*Plasmodium* antibody detection by ELISA in 2,515 donors showed a high positivity rate with a prevalence of 73.9% (1,859/2,515) (95% CI: 72.1-75.6%). Equivocal results were found in 13.1% of samples (329/2,515), and there were no detectable antibodies in 327 donors (13.0%, 95% CI: 11.7-14.4%) (Table 6B). Antibody prevalence differed significantly according to the season (P <0.05) (Table 7 and Figure 2).

**Table 6:** Comparison of microscopy with pLDH ELISA (A) and antibody ELISA (B) in blood donors

**A**		**pLDH ELISA**			
		**Positive**	**Equivocal**	**Negative**	**TOTAL**
	Positive	230	64	1	295
**Microscopy**	Negative	736	558	926	2,220
	TOTAL	966	622	927	2,515
**B**		**Antibody ELISA**			
		**Positive**	**Equivocal**	**Negative**	**TOTAL**
	Positive	231	34	30	295
**Microscopy**	Negative	1,628	295	297	2,220
	TOTAL	1,859	329	327	2,515

**Table 7:** **Positivity rate of pLDH ELISA and antibody ELISA tests according to infecting donors by *Plasmodium *during the seasons, long rainy season (LRS), short dry season (SDS), short rainy season (SRS), and long dry season (LDS)**

		**Season**				
		**LRS**	**SDS**	**SRS**	**LDS**	TOTAL
**pLDH ELISA**	**POS**	142 (24.1%)	206 (50.3%)	323 (51.3%)	295 (33.3%)	966 (38.4%)
**Antibody ELISA**	**POS**	453 (76.7%)	288 (70.1%)	368 (58.4%)	751 (84.7%)	1.859 (73.9%)

### pLDH prevalence in Benin blood donors

pLDH antigen was found in 38.4% of blood donors (966/2,515) (95% CI: 36.5-40.3%), with equivocal results (n=622/2,515, 24.7%) considered as positive for the calculation. The prevalence of pLDH antigen confirmed by microscopy was 230/966 (23.8%). The only sample that was positive by microscopy but negative by pLDH detection was a *P. malariae* infection case, notably a malaria-antibody positive sample (Table 6A).

pLDH antigen prevalence varied significantly according to the season (P < 0.05) (Table 7 and Figure 2). Interestingly enough, 25.1% of the blood donors with equivocal pLDH index were microscopically negative. However, a similar percentage was observed in microscopically positive blood donors, suggesting that the grey zone was correctly set up for pLDH (Table 8). The absence of antibodies in the blood of patients with detectable parasitaemia was noted in 30 cases (30/295; 10.2%), but 20 of them tested positive when using the pLDH ELISA. In 744/2,515 (29.6%) cases, both antibody and antigen were present in samples, and 199 samples were positive (n=112) or equivocal (n=87) for pLDH and negative for antibodies (Table 9).

**Table 8:** Distribution of pLDH ELISA in microscopically positive and negative blood donors

**pLDH ELISA index**	**Positive donors**		**Negative donors**	
	**Frequency**	**Percent (%)**	**Frequency**	**Percent (%)**
>20	45	15.3	2	0.01
10-20	24	8.1	2	0.01
1-10	161	54.6	737	33.2
0.7-0.99	64	21.7	558	25.1
Negative	1	0.3	921	41.5
TOTAL	**295**		**2,220**	

**Table 9:** Antibody ELISA and pLDH ELISA comparative results for Benin blood donors

		**pLDH ELISA**			
		**Positive**	**Equivocal**	**Negative**	**Total**
**Antibody ELISA**	Positive	**744**	442	673	1,859
	Equivocal	110	94	126	329
	Negative	112	87	128	327
	TOTAL	966	622	927	2,515

## Discussion

In this study, a commercialized pan *Plasmodium* LDH ELISA was shown to exhibit optimal performances as to both PPV and NPV. The assay was able to detect one parasite per μL, which is the lowest detectability reported in the scientific literature to date. This detection threshold is much lower than that of the already commercialized rapid test [28], which is estimated at 50 parasites/μL. An ELISA-based assay would theoretically lower that detectability. Indeed, another ELISA assay based on HRP2 detection was able to detect 11.7 parasites/μL, which is equivalent to 6 ng/ml of r*Pf*HRP2 [29]. The authors of this study conducted in Kenya reported that the microscopic detection limit was three parasites/μL, with a lower reproducibility. The ELISA assay, which is able to detect *in vitro* lower amounts of *P. falciparum* and other species, would thus be an interesting tool to detect the presence of *Plasmodium* in a given blood donor population. However the reagent failed to detect seven cases of *P. ovale* out of 19 cases. This defect could due to the incapability of the assay to detect the pLDH from some strains of *P. ovale*[30]. The use of a commercialized pan *Plasmodium* LDH ELISA was evaluated in a large blood donor population from southern Benin. The test results were compared to those obtained using microscopy and to those pertaining to malaria-antibody prevalence. The study aimed to determine whether pLDH detection was sensitive enough to identify low parasitaemia levels, which are frequently the case in asymptomatic malaria blood donors, and to assess the prevalence of positive pLDH blood donations in southern Benin.

This study enrolled 2,515 blood donors from six southern Benin departments, with similar malaria epidemiological features. These departments exhibit a wet tropical climate, with two rainy seasons and two dry seasons. In these regions, malaria transmission is permanent, with an increase during the rainy season [16]. The most transmitted species are *P. falciparum* (97.10%) and *P. malariae* (2.00%) [15,31]. The figures reported in the literature are similar to those found in this study, with a 95.0% prevalence of *P. falciparum* and 4.7% prevalence of *P. malariae*. The overall prevalence of asymptomatic *Plasmodium* carriage here was 11.72%. *Plasmodium falciparum* parasite density proved to be very low and highly variable. Two-third of these positive donors had a parasite density between seven and 100 parasites per μL. Reported prevalence rates in Benin appear to be similar to those reported by some authors in neighbouring Nigeria, 10.2% [32], or in Ghana, 10.20%. [33]. In this study, *P. falciparum* was identified in all infected cases, 5.0% of which were mixed *P. malariae* infections*.* These figures are in line with the reported presence of *P. falciparum* in all infected cases and the 2.3% rate of mixed infection in Nigeria [3]. However, these results differ from those of other authors who reported higher parasite prevalence rates, namely 50.7% in Burkina Faso [34], 55% in Nigeria [13,23], and 33.52% in Benin [4].

The study findings highlighted a higher infection rate in male blood donors compared to female ones (85.42 *vs* 14.6%). In a study conducted in Colombia [35], males were also found to be more infected with *Plasmodium ssp.* than females, 59 and 41%, respectively. Nevertheless, in a 1994 publication by Vlassoff *et al.*[36], Ghanaian women were found to have higher infection rates than Ghanaian men.

According to the study, the risk of *Plasmodium* transmission through blood transfusion is accounted for by the persistence of malaria parasites in blood. Canonical knowledge indicates that *P. falciparum* may persist in blood for one year, *P. ovale* for three years, and *P. malariae* for even longer time periods before causing malaria [20,32]. Similarly, the fact that malarial parasites may survive in RBCs at refrigerator temperatures (2-6°C) for days or weeks leads to the original exclusion of all blood donors who could represent a potential risk in TTM [6,7]. This observation supports some authors’ suggestions, namely to implement *Plasmodium* screening prior to blood donation in Africa [3,37].

In non-malaria-endemic area, the malaria antibody detection test will result in rejecting blood donation in case of positivity. However, in malaria-endemic countries, such as Benin, this tool is useless. Indeed, malaria antibody prevalence was 87% (74.0% positive and 13% equivocal) in the study’s blood donor populations. This prevalence was similar to that reported for Senegal, with 65.33% being positive and 6.86% equivocal [19]. Therefore, this high antibody seroprevalence in blood donors invalidates the marker’s use for detecting *Plasmodium-*infected blood donations. It is interesting to note that detectable *Plasmodium* parasites were found in blood donors but without antibodies, suggesting an acute infection.

In this study, 38.4% of the blood donors were tested positive for pLDH antigen, with a 4.2% rate of equivocal results. The pLDH antigen detection results differed from those found by Diop *et al.* in Senegal, who reported a detection rate of 0.53% for pLDH, with a 2.23% rate of doubtful cases [19]. However, the malaria epidemiology in Senegal is highly different from that in Benin. The low parasitaemia level may escape microscopic examination, while being dependent on the reader’s skill. The pLDH ELISA exhibited a detectability threshold lower than that of the other methods, with the exception of PCR. This finding suggests that pLDH detection could be a valid tool for blood bank settings in order to identify blood donation with a detectable parasitic load of at least one parasite per μL.

The questionnaire’s effectiveness helped to identify certain risk factors associated with pLDH antigen carriage, such as blood donations during high transmission periods. Based on the study results it was noticed that seasons influenced both microscopic and pLDH prevalence. Others factors likely impact these markers, such as the malaria prevention method used by blood donors, i.e. drugs, herbal teas, etc. The limits of selecting blood donors through medical questionnaires were highlighted by the study findings, as 38.4% positive donors for ELISA pLDH had met the selection criteria and gave their blood. It is, therefore, logical to propose implementation of *Plasmodium* screening in blood donation, which was not performed in Benin due to costs and lack of technique reliability, still keeping in mind that blood donors who report an undiagnosed and untreated febrile illness consistent with malaria should be deferred until asymptomatic and off the treatment. In a recent cost effectiveness analysis study, Rajab *et al.*[38] concluded that pretransfusion screening would be less costly than the recommended artemisinin-based combination used for prophylaxis in blood recipients, especially children and pregnant women.

## Conclusion

Malaria and transfusion is a “neglected subject coming back to the fore front”, as recently discussed by Allain [39]. The microscopy and antibody detection techniques are not suited for mass screening in a blood transfusion centre in endemic areas. Based on this study results, the pLDH antigen detection ELISA for *Plasmodium* could be an interesting tool for blood donation qualification in order to ensure blood safety in malaria-endemic areas. One of the test’s advantages is that false-positives are exceptional, unlike the HRP2 (histidine-rich-protein)-based tests, which can remain positive for two weeks after parasite clearance. HRP2 may also be absent in malaria infection cases. pLDH antigen ELISA detection in the blood is efficient when there is a parasite [40]. Some pitfalls were observed in the study. In some *P. ovale* infection cases the assay failed to detect them. Similarly, if the donors had taken self-treatment measures prior to blood donation, malaria infection was masked and pLDH detection failed. In conclusion, routine screening of all donated blood would prevent infected blood donation and increase the donation’s safety for fragile recipients, such as children and pregnant women. It would also be instrumental in reducing unnecessary medical treatments in recipients and contribute to lower *Plasmodium* resistance. The main problem remains the feasibility of rejecting positive donations in term of blood availability in endemic area where blood transfusion needs are presently increasing.

## Competing interests

The authors declare that they have no competing interests.

## Authors’ contributions

PSA, CDL, AS and EC designed the study, and performed the acquisition, the analysis and the interpretation of data. NWC, SP, TA, CDA, LA and AB were involved in the acquisition, the analysis and interpretation of data. All authors have given a final approval to the manuscript.
